# The Digital Age of Aging: Health through Telehealth, Digital Literacy, and Lifestyle Interventions

**DOI:** 10.1093/geroni/igaf122.1141

**Published:** 2025-12-31

**Authors:** Su-I Hou

## Abstract

This symposium explores digital health innovations for aging populations, addressing key challenges in accessibility, literacy, and engagement. Through real-world applications and research, we aim to identify best practices for integrating digital technologies into aging care and public health strategies. Drs. Park and Hou (UCF) will analyze telehealth adoption patterns using the 2022 Health Information National Trends Survey (HINTS). Findings reveal significant disparities in access, particularly among older adults, men, and rural populations, underscoring the need for broadband expansion and digital literacy initiatives. Dr. Savela (UEF) will examine digital skills and health literacy gaps among older caregivers using the Survey of Health, Ageing and Retirement in Europe (SHARE). Results highlights socioeconomic drivers of digital inequalities and the need for targeted interventions. Dr. Hou (UCF and UEF) will evaluate digital tools in dementia prevention within WW-FINGERS, the first global network of multidomain lifestyle intervention trials across 70 countries. The integration of digital tools in seven trials underscores their feasibility and the need for tailored strategies across diverse cognitive and social settings. Dr. Dino (UCF) will present findings from the MOVE Project, which leverages mixed reality (MR) to enhance older adults’ physical activity. Insights reveal ways to optimize MR-based interventions for engagement and usability. Dr. Thiamwong (UCF) will explore how digital health technologies, including wearable sensors and mobile apps, enhance physical function, motivation, and mental health in low-income older adults. Collectively, these studies underscore the potential of digital health to reduce disparities, foster engagement, and promote healthy aging. This is a collaborative symposium between the International Comparisons of Healthy Aging and Technology and Aging Interest Groups.

